# Advances in Using MRI to Estimate the Risk of Future Outcomes in Mental Health - Are We Getting There?

**DOI:** 10.3389/fpsyt.2022.826111

**Published:** 2022-04-12

**Authors:** Aleix Solanes, Joaquim Radua

**Affiliations:** ^1^Imaging of Mood- and Anxiety-Related Disorders (IMARD) Group, Institut d’Investigacions Biomèdiques August Pi i Sunyer (IDIBAPS), Barcelona, Spain; ^2^Department of Psychiatry and Forensic Medicine, School of Medicine, Autonomous University of Barcelona, Barcelona, Spain; ^3^Early Psychosis: Interventions and Clinical-detection Lab, Institute of Psychiatry, Psychology and Neuroscience, King’s College London, London, United Kingdom; ^4^Department of Clinical Neuroscience, Stockholm Health Care Services, Stockholm County Council, Karolinska Institutet, Stockholm, Sweden

**Keywords:** neuroimaging, machine-learning (ML), risk estimating, schizophrenia, magnetic resonance imaging (MRI)

## Introduction

Schizophrenia is a mental disorder among the leading disabling conditions worldwide (1). It affects approximately 20 million people and increases 2–3 times the probability of dying early and decreases the life expectancy by about 20 years (2). Its onset is typically in adolescence or early adulthood (3). Relevantly, prolonged untreated psychosis leads to poorer outcomes (4), so detecting the disorder early could considerably improve their lives.

Neuroimaging studies have found that individuals with schizophrenia have smaller volumes in the hippocampus, amygdala, thalamus, nucleus accumbens, intracranial space, and larger pallidum and ventricle volumes (5–9).

Traditionally, most neuroimaging studies in psychiatry relied on mass-univariate statistical approaches. For example, techniques like voxel-based morphometry (VBM) let researchers assess voxel-wise differences in regional volume or tissue composition based on estimates of tissue probability. These approaches are helpful to detect group differences. Still, they cannot yield predictions (e.g., diagnosis, outcome, etc.) at the individual level. In recent years, the interest in machine-learning methods in neuroimaging has increased due to its capability to handle high-dimensional data and perform predictions at a single-subject level.

The use of machine learning algorithms such as Support-Vector Machines (10), regularized regression (11, 12), Random Forest (13), or more recently, Deep Learning (14) to different neuroimaging modalities has expanded the possibilities of brain imaging data much beyond the traditional case-control group comparisons.

For example, many researchers aimed to use MRI-based data to detect mental disorders. Most focused on classifying each MRI scan as being from a patient or a healthy control (15–17). Still, others attempted to classify each MRI scan as being from a patient with one or another mental disorder (18–20). A recent systematic review summarized the classification performance between patients with schizophrenia and healthy control reported by various studies. High-performance prediction accuracy of >70% was reported in 40 of 41 studies using structural MRI, 35 of 40 using functional MRI scans, and 5 of 5 using diffusion-weight MRI (21). While these prediction rates may seem impressive, most of these diagnostic tools have not been integrated into clinical practice.

Less machine learning research has been done in detecting subjects at high risk of developing future outcomes in schizophrenia (22, 23). This is unfortunate because early detection could delay or even prevent severe future consequences (24). Some studies used clinical data, such as the presence of manic and negative symptoms (25–27), the diagnosis at onset together with other sociodemographic and clinical scales information (28, 29), or drugs use (28, 30, 31). Others used biological information such as blood-based biomarkers (32, 33), genetics data (34–36), or the combination of both clinical and biological data (37).

Finally, the use of MRI data to estimate the risk of different outcomes in schizophrenia has been even less explored. Only a few studies have taken this path, using as predictor variables the changes in brain volume during the 1st year after a first episode of psychosis (FEP) (38), brain gray matter MRI (39, 40), or surface-based data (40).

While results are still humble, we believe that these efforts are building a base on top of which future research will create valuable tools that will help the clinician. For example, these tools could help detect subjects at risk or predict the response to different treatments with the final aim to improve the patient wellbeing (41, 42).

The following section will describe which machine learning algorithms are currently used in neuroimaging. Then we will review which are the most common pitfalls and errors that we believe researchers should avoid. Finally, we will set our sights on the future, checking some of the promises of the latest algorithms and neuroimaging techniques.

## Common Machine Learning Algorithms

**FIGURE 1:**
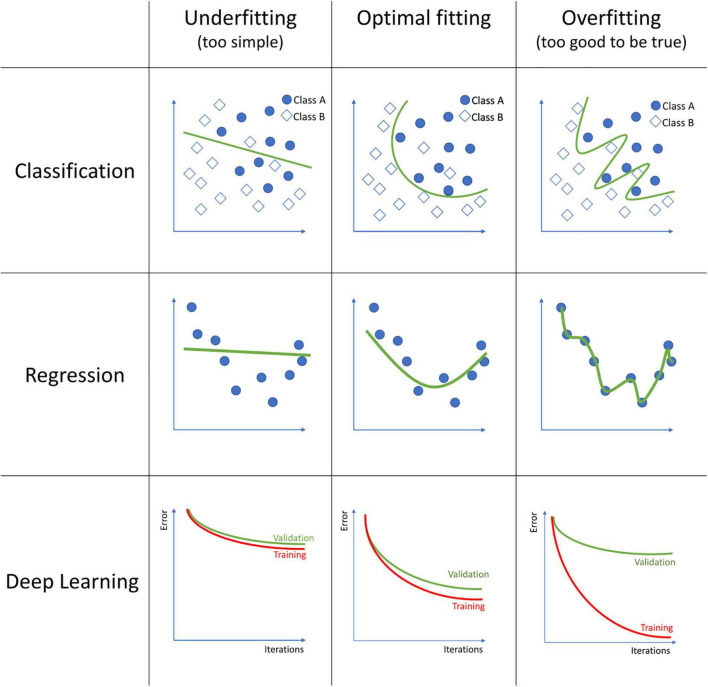
Examples of model underfitting, model fitting good, and a model overfitting to the data on classification, regression, and deep learning models. In classification, squares and circles represent two different classes. In Deep Learning, the figures represent the training and validation error over each iteration during the model training process.

The field of machine learning englobes an extensive list of algorithms, each with its strengths and weaknesses. Each of these algorithms should be tuned to obtain a trade-off between the model not fitting the data well enough, which is called underfitting, and excessive fitting the training data, called overfitting (see Figure 1). An underfitted model fails to capture the relationship between the MRI data and the outcome, for what it performs poorly even on the training data. Overfitting occurs when the model finds relationships between the MRI data and the outcome that are only based on particular, random details of the training data, and thus its performance is poor on new data. Over the vast selection of algorithms, we will review only the more common.

### Logistic Regression

Logistic Regression (LR) is a statistical model that fits the data to a logit or logistic function, an S-shaped sigmoid function. It calculates a binary dependent variable from one or more independent variables. Since neuroimaging results in a huge number of variables and many of them may be highly correlated, a technique called regularization is often used to reduce the complexity. Among the most used regularization algorithms in linear regression, we can find Least Absolute Shrinkage and Selection Operator (LASSO) (12), which eliminates non-important variables from the final model that do not contribute much to the prediction task, or Ridge (11), which instead of removing the variables, applies a penalty to some values that result in near-zero coefficients. A combination of both Ridge and LASSO also exists, which is called Elastic Net (43). This algorithm eliminates the coefficients of some variables and also reduces others close to zero.

Despite being a simple algorithm, it has been used to predict subjects at ultra-high risk and even performed better than more complex algorithms (44). Elastic Net was used to detect both functional and structural brain alterations in female patients with schizophrenia (45). Salvador et al. evaluated the discriminative power of some of the most commonly used algorithms on sMRI for prediction in psychosis. They systematically compared the different algorithms, and among them, Ridge, LASSO, and Elastic Net classifiers performed similarly, if not slightly better, than other classifiers (46).

### Support Vector Machines

Support Vector Machine (SVM) is a supervised discriminative classification method that creates hyperplanes for optimally separating the data into different groups that can be used for both classification or regression tasks. One strength of the algorithm is the ability to perform non-linear classifications. This is done by taking low dimensional input space data and convert them into a higher dimensional input space so that a hyperplane can separate the different classes. To improve the generalization of the model and avoid overfitting, a trade-off between maximizing the margin between the classes and minimizing the number of misclassifications has to be considered.

SVM algorithms emerged as powerful tools for finding objective neuroanatomical biomarkers since they can quite effectively handle high-dimensional data, consider inter-regional correlations between different brain regions, and make inferences at a single-subject level with a decent classification result (47).

It is probably one of the most commonly used machine learning classification algorithms in neuroimaging data. A common approach has been the use of SVM to classify subjects at ultra-high risk for psychosis from healthy controls (48, 49, 50). Due to its capability to deal with high-dimensional data, it has also been applied to classify between recent-onset depression and recent-onset psychosis using both neuroanatomical information and clinical data (51), to find neurocognitive subtypes based on cognitive performance and neurocognitive alterations in recent onset psychosis (52, 53), to identify schizophrenia patients based on subcortical regions (54) or functional network connectivity data (55). A multimodal approach combining structural MRI, diffusion tensor imaging, and resting-state functional MRI data was tested to classify patients with chronic schizophrenia vs. patients with FEP comparing different algorithms such as Random Forest (RF), LR, Linear Discriminant Analysis (LDA), and K-Nearest Neighbor classification (KNN), and SVM, resulting in the latter as the best performing one (56). Steardo et al. in a recent systematic review, analyzed 22 studies using SVM on fMRI as biomarkers to classify between schizophrenia patients and controls, where 19 studies reported a promising >70% accuracy (57).

The popularity of SVM and the trade-off between performance and simplicity are its main strengths. It is essential to consider that its performance is highly dependent on the hyperparameters chosen, and these are usually tuned using a grid search method (58).

### Linear Discriminant Analysis

Similar to SVM, linear discriminant analysis involves transforming the dimensionality of the data. However, in LDA, the data is projected into a lower-dimensional space where the different data groups can be maximally separated using a non-linear kernel (59).

It has been used on classification tasks such as Recent-Onset Schizophrenia (ROS) vs. HC (60), patients with FEP vs. HC (61–63), or patients with SZ vs. HC (64–67) with accuracies over 70%. Winterburn et al. used three independent datasets to validate the discriminative power of LDA to classify patients with SZ from HC, using different neuroimaging data. They compared cortical thickness data, ravens maps, and modulated VBM, and resulted that using their larger dataset, the accuracy was slightly lower compared to previous lower sample dataset articles (68).

The main strength of LDA is the reduction of the overfitting problem and computational cost by reducing the dimensional space. Despite that, its major drawback is that it requires assuming that the covariance matrix in the groups of data is identical, which is rare in real-world data.

### Decision Trees and Random Forest

Decision trees are non-parametric supervised learning methods used for both classification and regression. They can predict values by learning simple decision rules inferred from the data features. These algorithms tend to overfit, and so, to not generalize well to new data. To overcome this limitation, a variation on this algorithm exists called Random Forests (RF). It is simply a collection of Decision Trees whose results are aggregated into one single final result in the end. RF incorporates interactions between predictors in the model, detecting both linear and non-linear relationships.

It has been used to classify groups, such as childhood-onset schizophrenia patients and healthy controls (69), or schizophrenia, bipolar disorder, and healthy controls (70, 71).

It is an algorithm that generally provides high accuracy and a balance between bias-variance trade-off. Its major drawbacks are that it tends to be computationally intense on large datasets. It can be difficult to interpret the results, as it is difficult to analyze all coefficients.

### Artificial Neural Networks and Deep Learning

Artificial Neural Networks (ANN) are a family of machine learning algorithms inspired by the brain’s biological functioning. Like in our brain, these algorithms have neurons that receive a signal, process it, send a signal to the following connected neuron, and so on until a final result is obtained. To adjust the learning capabilities of the model, each neuron and synapse can have weights to increase or decrease the strength of the signal. Neurons are aggregated into layers, and when the number of layers increases, the algorithm is then known as Deep Learning. These advanced models can extract complex latent features from minimally preprocessed original data through non-linear transformations. To avoid overfitting, a method called Dropout exists. Dropout is a regularization method that randomly ignores or “drops out” some layer nodes, which is similar to adding noise to the training process. This improves the generalization of the model and reduces overfitting.

Deep Learning is often used to classify, predict values or even detect or segment regions in the brain.

Deep Learning is a vast topic that has increased the performance in some classification/prediction problems due to finding complex patterns on highly complex data. Despite being widely used to perform automatic tumor (72) or multiple sclerosis lesion detection (73) in brain MR images, it is still not extensively used in mental health disorder detection or risk-estimation of outcomes.

To overcome the black-box problem in artificial neural networks, different approaches are being developed, such as creating heat maps using Layer-Wise Propagation to identify the more important features involved in the algorithm decision (74, 75).

### Libraries and Tools

Machine learning analyses involve choosing both the algorithm and its parameters. The research on machine learning applied to neuroimaging wills to boost the algorithm’s performance. Still, some research groups decided to make their models available to the broad research community as open-source tools to let others use these improvements. This also enables researchers without coding backgrounds to perform machine learning analyses and expand the applicability and the validation of these tools. Some of these open-source tools focused on MRI machine learning are listed here:

NeuroMiner^1^ : It is a free Matlab toolbox developed by Nikolaos Koutsoleris with support from the PRONIA project that provides machine learning methods for analyzing heterogeneous data, such as clinical, structural, genetic, and functional neuroimaging data. It is designed to be easy to use, and no coding skills are required. Different MRI preprocessing steps are integrated into the software pipeline. It can create models to be applied to new data. It is designed to be flexible across different machine learning methods and data types, and it lets model sharing through a collaborative model library.

MRIPredict^2^ : It is a free GUI-based tool and also an R library maintained by the IMARD Group at IDIBAPS, Hospital Clínic de Barcelona. Like NeuroMiner, it provides machine learning methods to predict diagnosis from structural MRI data and clinical information. It can create models to be applied to new data and makes it easy to analyze which variables are useful for prediction or classification. It can handle multisite data by ComBat harmonization (76), perform techniques like ensemble learning to improve the robustness of the results, or multiple imputations to handle missing fields data. In addition, it can also conduct other types of predictions and estimations, such as the risk of developing a specific outcome based on the time to the event (survival analyses). It also can be used as an R library.

Pronto^3^ : It is also a free GUI-based Matlab developed by a team of researchers led by Prof. Janaina Mourao-Miranda. It provides various tools and algorithms for easily conducting machine learning analysis on neuroimaging data. The software lets the user specify the steps of the analysis as a batch job, choosing among the different functions of the software per each step.

Despite all the different algorithms, systematic comparisons between standard algorithms in neuroimaging pointed out that the differences in predictive performance are more attributable to differences in feature vectors than to the algorithm by itself (47, 77).

## Common Pitfalls and Errors

### Reproducibility and Replicability

While the following definitions are not universal, reproducibility commonly refers to obtaining (approximately) the same results of an article using the same data and experimental procedures used in that investigation. In contrast, replicability commonly refers to obtaining consistent results using new data.

Neuroimaging studies typically involve many possible choices during imaging quality control, preprocessing, or statistical analyses. In addition, in machine learning techniques, each algorithm may have several parameters that authors can set, e.g., to find the best method for their analyses.

To achieve reproducibility, all brain imaging and machine learning choices should be detailed and shared by the authors. Ideally, papers should report all details and choices made during the study. In addition, making the models, code, and data publicly available should be standard practice to let third parties examine the analysis in-depth and let others reproduce the whole process applied. That said, data may often be not sharable for privacy reasons.

In contrast, to achieve replicability, independent replication studies should be encouraged. Unfortunately, one common pitfall that prevents the applicability of many of these complex algorithms is their low replicability, their lack of generalizability. Many are developed on small sample sizes or single-site datasets because acquiring neuroimaging data is time-consuming and costly. However, while models made with small datasets or single-site data may seem to perform well, this performance is based on overfitting and, thus, the models fail when applied to new data. Like in human learning, the more examples an algorithm sees, the more it will learn to extrapolate those results to new samples (78). For example, recently, some authors evaluated some published models using clinical and neuropsychological data to predict the transition to psychosis in subjects at clinical high-risk for psychosis. When applying those published methods to a new sample, previously published models failed to predict or showed a poor accuracy (79, 80).

It is not uncommon in machine learning to test a battery of classifiers and report the one with the best accuracy. However, science should avoid studies based on cherry-picking, meaning taking only results advantageous for our research question. In some cases, this can be helpful, but this may easily lead to data torturing.

### Bias

Creating and validating machine learning models involves several steps that, if not performed carefully, may introduce sources of biases.

For example, many papers use a two-part study. The first part refers to creating the model (e.g., selecting which features best predict the outcome). The second part refers to its validation (e.g., applying it to estimate how well the model works). However, if researchers use the same dataset to create the model and validate it, the estimated accuracy will be inflated (81). Therefore, using a different dataset on model creation (including any feature selection) and validation is mandatory to avoid this bias.

Another example. Due to the difficulty of obtaining large datasets, collaborations between different sites are common. However, machine learnings may “fraudulently” use the differences between sites to predict the outcome. Therefore, these potential effects of the site must be very carefully controlled. Ignoring them may yield an inflated accuracy, even when the models do not really predict (76, 82).

### Clinical Utility

A common concern of clinical practitioners is the dubious clinical utility of some machine learning studies (83, 84). On the one hand, machine learning in medical data has proven to be an impressive tool in replicating and automating human processes, such as computer-automated detection (CAD) of lesions on brain scans, body scans, or mammograms (85). However, on the other hand, studies such as detecting whether an MR image belongs to a patient or a healthy control may seem clinically useless (68). We knowledge that these studies are indeed valuable as a proof-of-concept. However, we should progressively ensure that clinicians find them helpful, i.e., that the question answered by the model aligns well with clinical needs.

In this regard, it is essential to keep a distinction between what is a “model” and what is a “tool.” A model may be necessary for further investigation or methodological purposes. Conversely, a tool should be helpful, feasible, and safe for clinical decision-making in real-world settings (84).

## Challenges and Latest Advancements

### Longitudinal Studies

Methods based on single time-point data can be helpful. Still, changes over time may provide relevant information to create models of what may happen (e.g., if the patient will respond to treatment or have a complication). For example, it is known that patients with a first episode of psychoses show a decrease over time in cortical gray matter when compared with healthy controls (86), or that progressive gray matter volume reduction in the superior temporal gyrus is associated to low improvement in positive psychosis symptoms (87). Having a dataset collected from hundreds or thousands of people with similar conditions over an extended period will enable more complex patterns to be found. These patterns will allow a better future outcome prediction. Therefore, longitudinal studies will be crucial in improving the reliability and performance of mental health decision-helper tools.

### Larger Datasets

One of the common first steps when preprocessing neuroimaging data is reducing dimensionality, using expert-designed feature selection or feature extraction. This process boosts the performance of algorithms, but it removes information from the input data. Conversely, modern algorithms like deep learning can use minimally preprocessed input data and take advantage of the subtle patterns usually withdrawn during preprocessing (88). However, although already used in some brain abnormality detection tasks, deep learning has not yet been extensively applied to detect early subjects at risk of developing a disorder or a relevant outcome. A critical reason for not using deep learning algorithms is that they require, in general, substantially larger datasets than other machine learning approaches.

Neuroimaging datasets tend to be hard to acquire. Still, emerging consortia, like the ENIGMA consortium,^4^ are already making it possible to conduct analyses on large datasets otherwise impossible to recruit (76). Indeed, a larger multisite sample not only improves the statistical power of the studies and allows the use of deep learning but also enhances the generalizability of the models to new data.

### New Algorithms

Algorithms and methods evolve every day, so maybe the best tool to detect subjects at risk is still testing. In this section, we will only scratch the surface and review some of the most promising methods in machine learning.

#### Self-Defining Algorithms

There are many possible machine learning algorithms to apply to a concrete question. The problem is which algorithm or hyperparameters are the best for each outcome. A new methodology called AutoML consists of techniques that can automatically select the appropriate model and its associated hyperparameters to optimize the performance and reliability of the resulting predictions (89). Having the algorithm self-define its characteristics can provide a human-agnostic model definition that is not prone to the biases and assumptions tied to each decision the expert makes when defining a model. It has already been tested in identifying digital phenotyping measures that are more relevant for negative symptoms in psychotic disorders successfully (90).

#### Combining Knowledge From Other Sources

In other domains, like in computer vision, large datasets exist for general purposes, such as ImageNet (91). But in neuroimaging is not so easy to achieve such a giant dataset. Here is where a technique called Transfer Learning appears. This approach can extract insights obtained in large general-purpose datasets and use that information to improve small dataset model creation (82). This technique has already been tested to improve Alzheimer’s disease classification (92). Still, to our knowledge, it has not been used in many other domains like risk estimation.

#### Explainable Artificial Intelligence

Novel algorithms like Deep Learning are usually considered “black boxes” because networks’ decisions are not easily interpretable by humans. Explainable Artificial Intelligence (XAI) seeks to provide an easily understandable solution. For example, in highly complex neural networks used for MRI-based classification, it is not easy to know which voxels have been used to classify between groups; XAI would provide a heat map indicating which were the more relevant zones or voxels used in classification, providing insights into how the network works (93). One approach is the layer-wise relevance propagation (LRP), which produces heatmaps of the contribution of each voxel to the final classification outcome at a single-subject level. When tested in Alzheimer’s disease, the voxels reported in the heatmap were concordant with zones associated with AD abnormalities in previous literature (74). It has also been applied to multiple sclerosis, where the lesions are distributed across the brain. The individual heatmaps corresponded to the lesions themselves and non-lesion gray and white matter areas such as the thalamus, which are conventional MRI markers (75). In a study where authors used texture feature maps for classifying participants with SZ, MD patients, and HC, LRP showed which zones contributed to the classification of the deep learning algorithm (94). Another interesting approach to determine which regions contribute the most to classification consists of substitute brain regions by healthy ones generated using variational autoencoders and then see how performance changes (95).

Having tools understandable for humans would make it easier for the researchers, clinicians, and general population to believe in them.

#### Federated Learning

One obstacle in data sharing for creating larger datasets in MRI is the concern about privacy and confidentiality. And another limitation is that despite having large imaging databases, many images have few labels and therefore do not allow the model to learn much. A trained radiologist must inspect the images to annotate the labels, which can be time-consuming. Both problems can be solved using federated learning since it trains algorithms across multiple health care sites. An algorithm is provided to all the centers and is applied locally at each site. Once the algorithm extracts the information, this knowledge is put together. Using this approach, no private data is shared, and centers can help in the process even if their labeled database is small. Federated learning is a promising technique that upholds patients’ privacy and eases the cooperation between health care centers (96).

### A Multimodal Approach

Schizophrenia and other mental disorders are known to be caused by a combination of genetic, anatomical, and environmental factors. Therefore, predictions of future outcomes or the early detection of subjects at risk can profit from multimodal approaches, e.g., combining genetic and neuroanatomical factors. Many studies are indeed already using a multimodal approach (97). However, the main problem is that it is still unclear which combination of factors best predict the outcome and how to combine them.

## Discussion

This article describes some common techniques and algorithms used in neuroimaging machine learning research. It also reviews some errors and pitfalls that may affect their models’ replicability and clinical utility. Finally, it scratches on the surface of some of the topics that can be relevant shortly. One of them may be focusing on acquiring longitudinal data, which may address clinically relevant questions (e.g., whether a patient will respond to one or other treatment). Another topic may be related to emerging promising techniques. For instance, human-agnostic model definition algorithms could provide new assumption-free methods not relying on human choices on algorithm definition. Transfer Learning algorithms could allow using algorithms that have been intensively trained in other fields. Other algorithms may overcome the “black box” machine learning problem. Or, on another note, Federated Learning may ease collaboration between centers, achieving the larger sample sizes required for machine learning.

## Author Contributions

AS and JR worked equally on the drafting and revision of the manuscript. Both authors contributed to the article and approved the submitted version.

## Conflict of Interest

The authors declare that the research was conducted in the absence of any commercial or financial relationships that could be construed as a potential conflict of interest.

## Publisher’s Note

All claims expressed in this article are solely those of the authors and do not necessarily represent those of their affiliated organizations, or those of the publisher, the editors and the reviewers. Any product that may be evaluated in this article, or claim that may be made by its manufacturer, is not guaranteed or endorsed by the publisher.

## Footnotes

1
http://proniapredictors.eu/neurominer/

2
https://mripredict.com

3
http://www.mlnl.cs.ucl.ac.uk/pronto/

4
http://enigma.ini.usc.edu/
